# Triggering Receptor Expressed on Myeloid Cells (TREM)-2 Impairs Host Defense in Experimental Melioidosis

**DOI:** 10.1371/journal.pntd.0004747

**Published:** 2016-06-02

**Authors:** Tassili A. F. Weehuizen, Tijmen J. Hommes, Jacqueline M. Lankelma, Hanna K. de Jong, Joris. J.T.H. Roelofs, Alex F. de Vos, Marco Colonna, Tom van der Poll, W. Joost Wiersinga

**Affiliations:** 1 Center for Infection and Immunity Amsterdam (CINIMA), Academic Medical Center, Amsterdam, the Netherlands; 2 Center for Experimental and Molecular Medicine (CEMM), Academic Medical Center, Amsterdam, the Netherlands; 3 Department of Pathology, Academic Medical Center, Amsterdam, the Netherlands; 4 Department of Pathology, Washington University in St. Louis, St. Louis, Missouri, United States of America; 5 Department of Medicine, Division of Infectious Diseases, Academic Medical Center, Amsterdam, the Netherlands; Institut Pasteur, FRANCE

## Abstract

**Background:**

Triggering receptor expressed on myeloid cells (TREM) -1 and TREM-2 are key regulators of the inflammatory response that are involved in the clearance of invading pathogens. Melioidosis, caused by the "Tier 1" biothreat agent *Burkholderia pseudomallei*, is a common form of community-acquired sepsis in Southeast-Asia. TREM-1 has been suggested as a biomarker for sepsis and melioidosis. We aimed to characterize the expression and function of TREM-1 and TREM-2 in melioidosis.

**Methodology/Principal Findings:**

Wild-type, TREM-1/3 (*Trem-1/3*^*-/-*^) and TREM-2 (*Trem-2*^*-/-*^) deficient mice were intranasally infected with live *B*. *pseudomallei* and killed after 24, and/or 72 h for the harvesting of lungs, liver, spleen, and blood. Additionally, survival studies were performed. Cellular functions were further analyzed by stimulation and/or infection of isolated cells. TREM-1 and TREM-2 expression was increased both in the lung and liver of *B*. *pseudomallei*-infected mice. Strikingly, *Trem-2*^*-/-*^, but not *Trem-1/3*^*-/-*^, mice displayed a markedly improved host defense as reflected by a strong survival advantage together with decreased bacterial loads, less inflammation and reduced organ injury. Cellular responsiveness of TREM-2, but not TREM-1, deficient blood and bone-marrow derived macrophages (BMDM) was diminished upon exposure to *B*. *pseudomallei*. Phagocytosis and intracellular killing of *B*. *pseudomallei* by BMDM and alveolar macrophages were TREM-1 and TREM-2-independent.

**Conclusions/Significance:**

We found that TREM-2, and to a lesser extent TREM-1, plays a remarkable detrimental role in the host defense against a clinically relevant Gram-negative pathogen in mice: TREM-2 deficiency restricts the inflammatory response, thereby decreasing organ damage and mortality.

## Author Summary

Triggering receptor expressed on myeloid cells (TREM)-1 and -2 are receptors on immune cells that act as mediators of the innate immune response. It is thought that TREM-1 amplifies the immune response, while TREM-2 acts as a negative regulator. Previously, we found that TREM-1 is upregulated in melioidosis patients. In contrast, nothing is known on TREM-2 expression and its role in melioidosis. In this study we examined the expression and functional role of both TREM-1 and -2 in a murine melioidosis model. We found that TREM-1 and-2 expression was upregulated during melioidosis. Using our experimental melioidosis model, we observed that *Trem-2*^*-/-*^ mice were protected against *B*.*pseudomallei*-induced lethality. *Trem-2*^*-/*-^ mice demonstrated reduced bacterial loads, inflammation and organ damage compared to wild-type mice in experimental melioidosis. Despite reduced bacterial dissemination of *B*.*pseudomallei* to distant organs in *Trem-1/3*^*-/*^ mice^*-*^, no differences in survival were found between *Trem-1/3*^*-/-*^ and wild-type mice during melioidosis. Lastly, we investigated cellular functions of TREM-1 and TREM-2 and found that TREM-2 deficiency led to decreased cellular responsiveness to *B*. *pseudomallei* infection. In conclusion, we found that TREM-2 plays an important role during experimental murine melioidosis. TREM-2-deficiency reduces inflammation and organ damage, thereby improving survival.

## Introduction

In sepsis, defined as a deregulated host response to a life-threatening infection, a careful balance between inflammatory and anti-inflammatory responses is vital [1–3]. Pathogen- or danger-associated molecular patterns are recognized by intracellular sensory complexes and cell surface receptors expressed on innate immune cells that can initiate the inflammatory and anti-microbial response. Well-known examples of these pattern recognition receptors (PRRs) are the Toll-like receptor (TLR), nucleotide-oligomerization domain-like receptor (NLR) and C-type lectin receptor (CLR) families [4]. A more recently discovered group of innate immune receptors are the membrane-bound triggering receptors expressed on myeloid cells (TREMs), which act as key modulators, rather than as initiators, of the inflammatory response [5–7].

TREM-1 and TREM-2 are the most studied members of the TREM-family, however their exact role in the pathogenesis of sepsis remains ill-defined. Upon recognition of partially still unspecified ligands, both receptors phosphorylate the adaptor molecule DNAX adaptor protein 12 (DAP12) after which the cellular response is initiated [8, 9]. Only recently, binding of TREM-1 to a complex of peptidoglycan recognition protein 1 (PGLYRP1) and bacterially derived peptidoglycan has been demonstrated [10]. TREM-1 is expressed on neutrophils and monocyte subsets [11] and amplifies pro-inflammatory TLR-mediated responses *in vitro* [12]. There are conflicting reports on the role of TREM-1 in *in vivo* infection models. TREM-1 deficiency impaired bacterial clearance in a model of *Klebsiella pneumonia*-induced liver abscess formation [13], pneumococcal [14] and *Pseudomonas (P*.*) aeruginosa* pneumonia [15]. However, blocking TREM-1 with an analogue synthetic peptide derived from the extracellular moiety of TREM-1 (LP17) actually improved survival during gram-negative sepsis [16] and endotoxaemia [17]. Interestingly, in a murine pneumonia model of *Legionella pneumonia* no impact of TREM-1 deficiency was found on bacterial clearance or neutrophil influx towards the primary site of infection [18].

TREM-2 is primarily expressed on macrophages, dendritic cells, microglia and osteoclasts [19–22] and has been suggested to bind to bacterial lipopolysaccharide (LPS) and lipotechoic acid [23]. In contrast to TREM-1, TREM-2 acts as a negative regulator of inflammatory responses in macrophages and dendritic cells [19, 21]. In addition, TREM-2 is involved in phagocytosis [24, 25] and killing of bacteria by macrophages [26]. Blocking TREM-2 *in vivo* by a recombinant protein in a polymicrobial sepsis model revealed that TREM-2 is required for bacterial clearance and improves survival [27]. In contrast, TREM-2 plays a detrimental role during pneumococcal pneumonia [25].

Melioidosis, considered to be an illustrative model for Gram-negative sepsis, is caused by the Tier 1 biological treat agent *Burkholderia pseudomallei* [28, 29]. Melioidosis is characterized by pneumonia and abscess formation and an important cause of community-acquired sepsis in Southeast Asia and Northern Australia [28]. The high mortality rate, that can approach 40%, and the emerging antibiotic resistance of *B*.*pseudomallei* [30] emphasize the need to better understand the pathogenesis of melioidosis, which could ultimately lead to novel treatment strategies. We previously found increased soluble (s) TREM-1 plasma levels and TREM-1 surface expression on monocytes of patients with melioidosis [31], suggesting an important role for TREM-1 in the host defense against *B*. *pseudomallei*. Treatment with a peptide mimicking a conserved-domain of sTREM-1 partially protected mice from *B*. *pseudomallei* induced lethality [31].

In this study we now examine the role of TREM-1 and TREM-2 during experimental melioidosis, utilizing recently generated *Trem-1/3*-deficient (*Trem-1/3*^*-/-*^) [15] and *Trem-2*-deficient (*Trem-2*^*-/-*^) mice [19] to determine their contribution to the host response against *B*. *pseudomallei*. We hypothesized that TREM-1 deficiency would decrease inflammation and improve survival during murine melioidosis while TREM-2 deficiency would instead lead towards increased inflammation and a worsened survival. Unexpectedly however, we found that TREM-2, but not TREM-1, plays an important detrimental role during melioidosis. TREM-2 deficiency improves survival of *B*. *pseudomallei* infected mice, by limiting inflammation and organ damage. These data identify TREM-2 as a potential treatment target for sepsis caused by *B*. *pseudomallei*.

## Materials and Methods

Detailed methods are provided in the online supplement (S1 Appendix).

### Ethics statement

The Animal Care and Use of Committee of the University of Amsterdam approved all experiments (DIX102273), which adhered to European legislation (Directive 2010/63/EU).

### Mice

Pathogen-free 8- to 10-week-old male wild-type (WT) C57BL/6 mice were purchased from Charles River (Leiden, The Netherlands). *Trem-1/3*^*-/-*^ [6, 14] and *Trem-2*^*-/-*^ [19] mice were backcrossed >97% to a C57BL/6 genetic background.

### Experimental infection and assays

*B*. *pseudomallei*, derived from our aliquoted frozen stock, was grown to log-phase and further diluted in sterile PBS (1x). Experimental melioidosis was induced by intranasal inoculation with 5 × 102 colony forming units (CFU) of *B*. *pseudomallei* strain 1026b (a clinical isolate) as described [32–34]. For survival experiments mice were observed 4–6 times daily, up to 14 days post-infection. Sample harvesting, processing, and determination of bacterial growth were performed as described in detail in the S1 Appendix[33, 34]. All work concerning live *B*. *pseudomallei* was performed in a (A)BSL III facility.

Chemo- and cytokine levels were determined in plasma, lung and liver. Distant organ damage was more closely assessed by plasma transaminases, lactate dehydrogenase (LDH) and blood urea nitrogen (BUN) levels.

### TREM-1 and TREM-2 expression

Total RNA was isolated using the Isolate II RNA mini kit (Bioline, Taunton, MA, USA), treated with DNase (Bioline) and reverse transcribed using an oligo(dT) primer and Moloney murine leukemia virus RT (Promega, Madison, WI, USA). Primers and RT-PCR conditions can be found in the supplemental data. Data were analyzed using the comparative C*t* method.

### (Immuno)histology

Paraffin-embedded 4-μm lung, liver and spleen sections were stained with haematoxylin and eosin and analyzed for inflammation and tissue damage, as described previously [14, 34]. Granulocyte (Ly6G) staining was done exactly as described previously [35].

### Whole blood and macrophage stimulation

Whole blood, alveolar macrophages (AM) and bone-marrow derived macrophages (BMDM) were harvested from naïve WT and *Trem1/3*^*-/-*^ and *Trem-2*^*-/-*^ mice as described [34, 36, 37] and stimulated overnight with either medium, ultrapure LPS (Invivogen, San Diego, CA, USA) or *B*. *pseudomallei* (107 CFU/ml or MOI of 50), after which supernatant was harvested and stored at -20°C until assayed for TNFα.

### Phagocytosis and bacterial killing

Phagocytosis was determined as described previously [38]. In brief, AM and BMDM (5x 104 cells/well) were incubated with or without heat-inactivated FITC-labelled *B*. *pseudomallei* (MOI 50) for 60 and/or 120 minutes at 37°C and 5% CO2 air and internalization was measured directly after collection by flow cytometry.

Bacterial killing was evaluated as described [36, 39]. In short, BMDM were incubated with to log-phase grown *B*. *pseudomallei* (MOI 30) for 20 minutes at 37°C in 5% CO2 air, after which they were washed and incubated with kanamycin 250 μg/ml for 30 minutes at 37°C in 5% CO2 air (this point was taken as time zero) [36]. At designated time points the BMDM were washed and lysed and appropriate dilutions of these lysates were plated onto blood-agar plates and incubated at 37°C for 24–48 h before CFU were counted.

### Statistical analysis

Values are expressed as mean ± standard error of the mean (SEM). Differences between groups were analyzed by Mann-Whitney *U* test. For survival analysis, Kaplan-Meier analysis followed by log-rank test was performed. These analyses were performed using GraphPad Prism version 5.01. Values of *P*< 0.05 were considered statistically significant.

## Results

### Increased TREM-1 and TREM-2 expression in the lung and liver during experimental melioidosis

**Fig 1:**
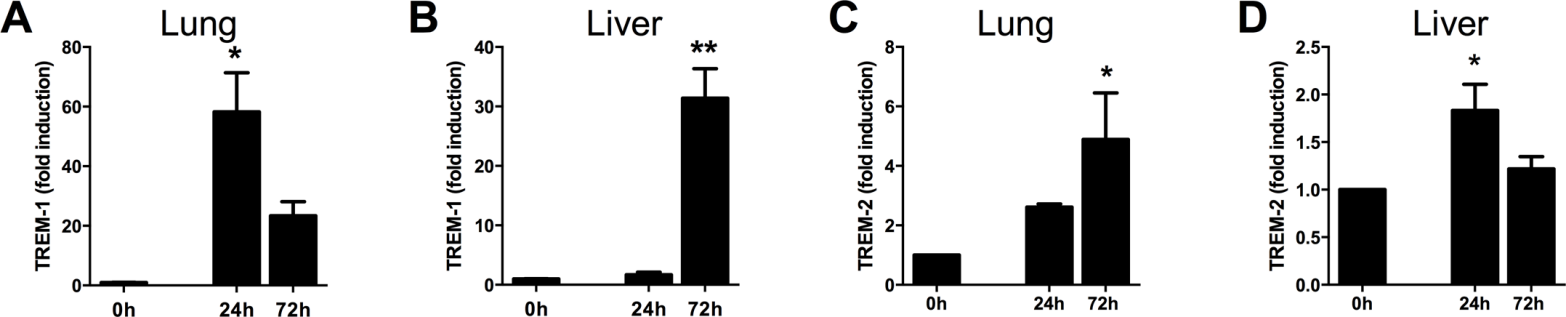
Increased TREM-1 and TREM-2 expression in experimental melioidosis. TREM-1 and TREM-2 mRNA expression was determined in wild type (WT) mice prior to infection or at 24 or 72h post-infection with 5 x 102 CFU *B*.*pseudomallei* intranasally. TREM-1 mRNA expression in lung (*A*) and liver (*B*) was determined. Likewise, TREM-2 mRNA expression was measured in lung (*C*) and liver (*D*) tissue. Data are presented as fold induction compared to the mRNA expression in uninfected mice (all RNA data are normalized to GAPDH). Data are mean ± SEM, n = 4–5 mice/group. * *P*< 0.05, ** *P* < 0.01, compared to gene-expression at t = 0h (Mann-Whitney *U* test).

Septic melioidosis patients present with pneumonia and bacterial dissemination to distant body sites [28, 40]. Since it is not feasible to study TREM-1 and TREM-2 mRNA expression at tissue level in these patients, we used our well-established murine model of pneumonia-derived melioidosis in which mice are intranasally infected with a lethal dose of *B*. *pseudomallei* [33, 34]. Mice were killed at 0, 24, and 72h after infection (i.e., directly before the first predicted death), and TREM-1/-2 mRNA expression was determined in lungs and livers. At baseline, TREM-1 and TREM-2 expression was low, corresponding with our previous data on sTREM-1 levels in melioidosis patients [31], TREM-1 was strongly up-regulated in lung and liver tissue (*P*<0.05 lung at 24h, *P*<0.01 liver at 72h; Fig 1A and 1B). TREM-2 mRNA expression was increased in experimental melioidosis as well (*P*<0.05 in both lung and liver; Fig 1C and 1D). The increase in both TREM-1 and TREM-2 expression was much more pronounced at the primary site of infection, the pulmonary compartment, when compared to the hepatic compartment.

### *Trem-2*^*-/-*^ mice, but not *Trem-1/3*^*-/-*^ mice, are protected from *B*. *pseudomallei*-induced mortality

**Fig 2:**
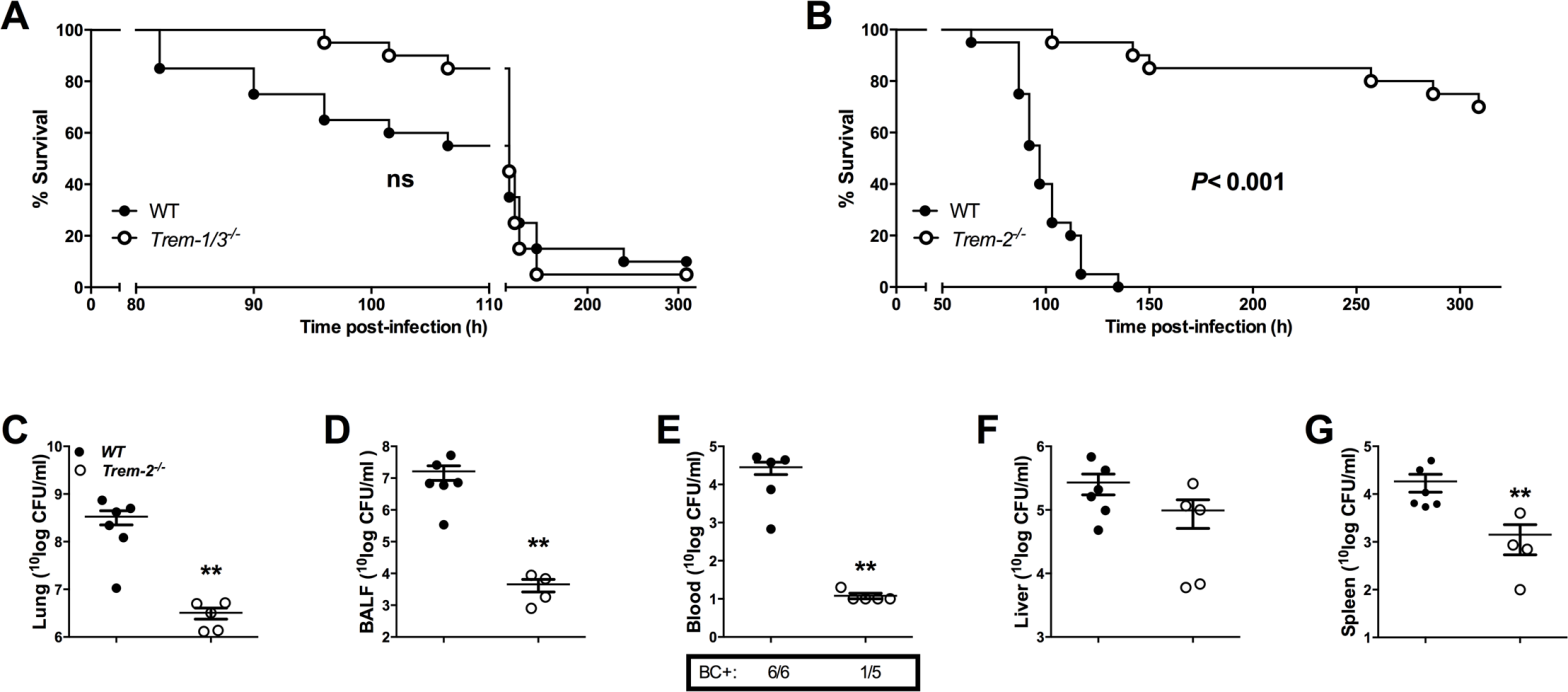
Survival of *Trem-2*^*-/-*^ mice, but not of *Trem-1/3*^*-/-*^ mice, is enhanced in experimental melioidosis. Survival was observed for every 4-6h, up to a maximum of 14 days after intranasal inoculation with 5 x 102 CFU *B*. *pseudomallei* in wild-type (WT; closed circles) and *Trem-1/3*^*-/-*^ mice (open circles; *A*). Similarly, survival of WT (closed circles) and *Trem-2*^*-/-*^ mice (open circles) was determined (*B*) (n = 20 per group). The *P* value indicates significance of the difference in survival between *Trem-2*^*-/-*^ and WT mice (Kaplan-Meier analysis, followed by a log-rank test). ns = not significant. In addition, WT (closed circles) and *Trem-2*^*-/-*^ mice (open circles) were infected with 5 x 102 colony forming units (CFU) of *B*. *pseudomallei* intranasally (n = 5–6 mice per group) and sacrificed 72 h post-infection, in order to determine bacterial loads in lung homogenates (*C*), broncho-alveolar lavage fluid (BALF) (*D*), whole blood (*E*), liver (*F*) and spleen (*G*). Data are expressed as mean ± SEM, n = 5-6/group. ** *P*< 0.01. BC+ denotes positive blood cultures (Mann-Whitney *U* test).

Having established that both TREM-1 and TREM-2 are highly up-regulated during melioidosis, we further investigated the involvement of these receptors in the outcome of melioidosis. Therefore, we infected WT, *Trem-1/3*^*-/-*^ and *Trem-2*^*-/-*^ mice intranasally with a lethal dose of *B*. *pseudomallei* and observed them for 14 days (Fig 2A and 2B). There was no significant difference in survival between *Trem-1/3* and WT mice following a lethal *B*. *pseudomallei* challenge: 95% of *Trem-1/3*^*-/-*^ and WT mice died within 6 days after inoculation (Fig 2A). Strikingly however, *Trem-2*^*-/-*^ mice were significantly protected: 70% of *Trem-2*^*-/-*^ survived until the end of the 14-day observation period while all WT mice died within 6 days (*P*< 0.001; Fig 2B).

### Enhanced bacterial clearance in *Trem-2*^*-/-*^ mice

To substantiate the finding that *Trem-2*^*-/-*^ mice are protected during melioidosis, we determined bacterial loads in lung and BALF as well as in blood, liver and spleen 72h post-infection. Relative to WT mice, *Trem2*^*-/-*^ mice displayed strongly reduced bacterial loads both at the primary site of infection (*P*<0.01 for lung and BALF; Fig 2C and 2D) as well as in distant organs and the systemic compartment (*P*<0.01 for blood and spleen; Fig 2E–2G). 72h post-infection 100% of WT but only 20% of *Trem2*^*-/-*^ mice had become bacteraemic. These findings indicate that TREM-2 plays a key deleterious role during experimental melioidosis by antagonizing bacterial clearance leading to increased dissemination of infection.

### *Trem-2*^*-/-*^ mice demonstrate reduced lung inflammation

**Fig 3:**
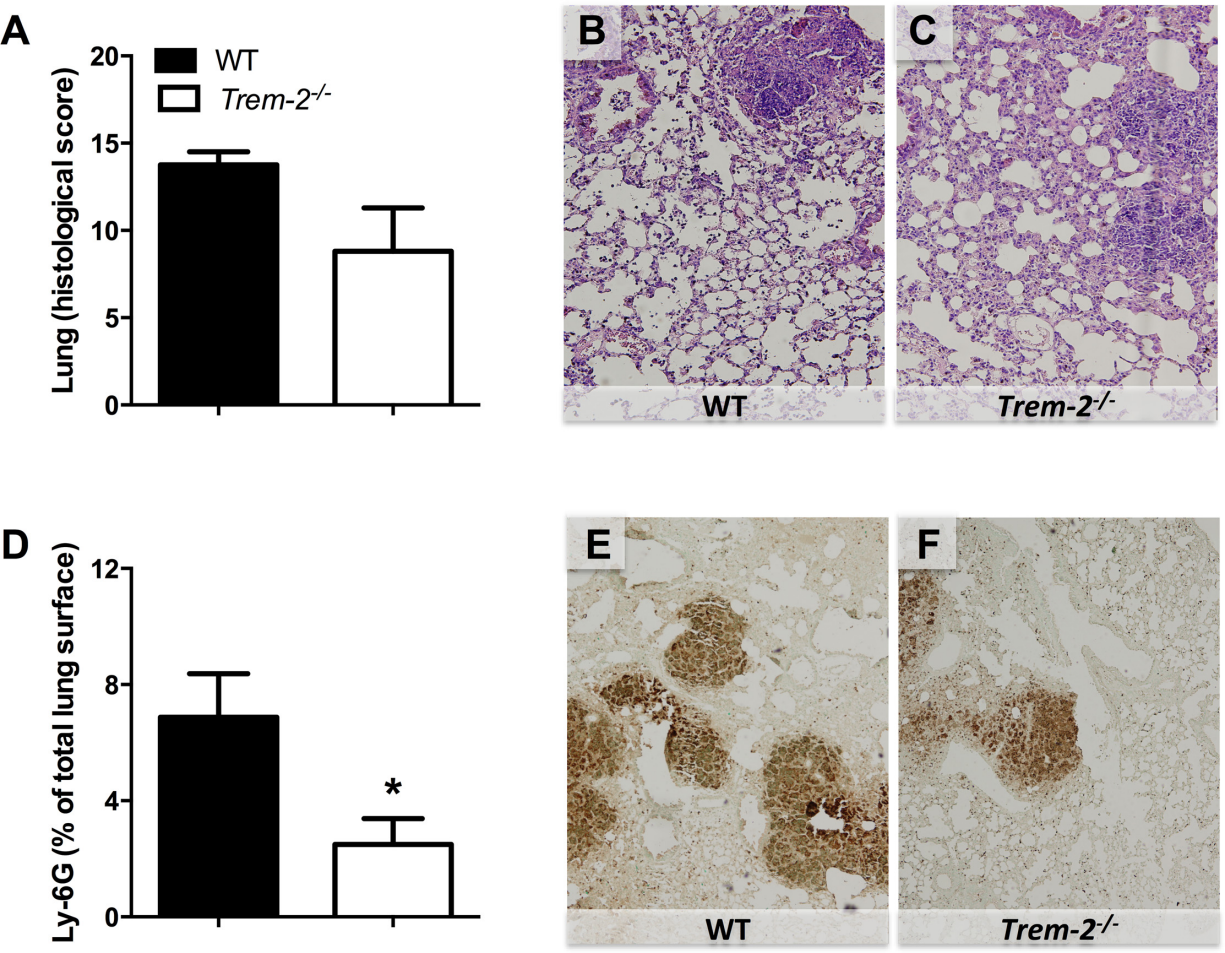
Reduced neutrophil influx in lungs of *Trem-2* ^*-/-*^ mice, without affecting lung pathology. Lung pathology was determined in wild-type (WT; black bars) and *Trem-2*^*-/-*^ mice (white bars) infected with 5 x 102 CFU *B*. *pseudomallei* at 72h post-infection as described in the Methods section (*A*). Representative lung slides of WT (*B*) and *Trem-2*^*-/-*^ mice (*C*) (original magnification 10x). Neutrophil influx was defined by Ly6G positivity (expressed as % of total lung surface; *D*). Representative photographs of Ly6G-immunostaining for granulocytes on lung slides of WT (*E*) and *Trem-2*^*-/-*^ mice (*F*) (original magnification 10x). Data are expressed as mean ± SEM, n = 5–6 mice per group per time point. * *P* < 0.05. (Mann-Whitney *U* test).

Since TREM-2 has been described as a negative regulator of inflammation [19, 20], we next assessed the inflammatory response in the pulmonary compartment. Therefore we studied the extent of inflammation in lung homogenates and BALF. We observed markedly decreased levels of pro-inflammatory cytokines TNF-α, IL-6, IL-1β and the chemokine KC in both lung homogenates and BALF of TREM-2 deficient mice compared to controls (*P*<0.01–0.05; Table 1). To further obtain insight into the involvement of TREM-2 in the inflammatory response following *B*. *pseudomallei* infection, we semi-quantitatively scored lung histology slides generated from *Trem-2*^*-/-*^ and WT mice. However, all mice displayed severe pulmonary inflammation and no differences were observed between the mouse strains (Fig 3A–3C). Neutrophil recruitment to the lung is an essential part of the inflammatory host response to melioidosis. Therefore, we determined the granulocyte influx into the pulmonary compartment by Ly6G-immunostaining in WT and *Trem-2*^*-/-*^ mice 72h post-infection with *B*. *pseudomallei* (Fig 3D–3F). This immunostaining recognizes Gr-1, that is granulocyte-specific, Corresponding to the diminished bacterial loads and decreased levels of cyto- and chemokines in lung tissue, a reduced influx of granulocytes in lungs of *Trem-2*^*-/-*^ mice was found (*P*<0.05, Fig 3D).

**Table 1:** Cytokine response in lung homogenates, BALF and plasma of WT and *Trem-2*^*-/-*^ mice during experimental melioidosis.

	T = 72h	
	WT	*Trem-2*^*-/-*^
**pg/ml**	**Lung homogenate**	
**TNF-α**	1680 ± 222	512 ± 156 **
**IL-6**	7025 ±1408	450 ± 66*
**KC**	59588 ± 9304	10580 ± 2233**
**IL-1β**	31292 ± 4975	1860 ± 516**
	**BALF**	
**TNF-α**	7054 ± 1578	1689 ± 171*
**IL-6**	18478 ± 4471	406 ± 204 **
**KC**	30702 ± 6626	1055 ± 454**
**IL-1β**	10469 ± 2424	217 ± 81**
	**Plasma**	
**TNF-α**	2324 ± 909	91 ± 19**
**IL-6**	2641 ± 526	31 ± 5**
**IL-10**	11 ± 3	5 ± 0**
**MCP-1**	1675 ± 453	11 ± 1**
**IFN-γ**	158 ± 62	17 ± 3**
**IL-1β**	1123 ± 303	125 ± 25*

Cytokine levels in lung homogenate, broncho-alveolar fluid (BALF) and plasma were measured after intranasal infection with 5 x 102 CFU wild-type *B*. *pseudomallei*. Wild-type (WT) and *Trem-2*^*-/-*^ mice were sacrificed 72 h after infection. Data are represented as means ± SEM (n = 5-6/group). TNF-α = Tumor necrosis factor-α; IL = Interleukin; MCP-1 = Monocyte Chemoattractant Protein-1; KC = Keratinocyte Chemoattractant; IFN-γ = Interferon-γ

* *P*< 0.05

** *P*< 0.01.

### Trem-2 deficiency leads to decreased distant organ injury during experimental melioidosis

**Fig 4:**
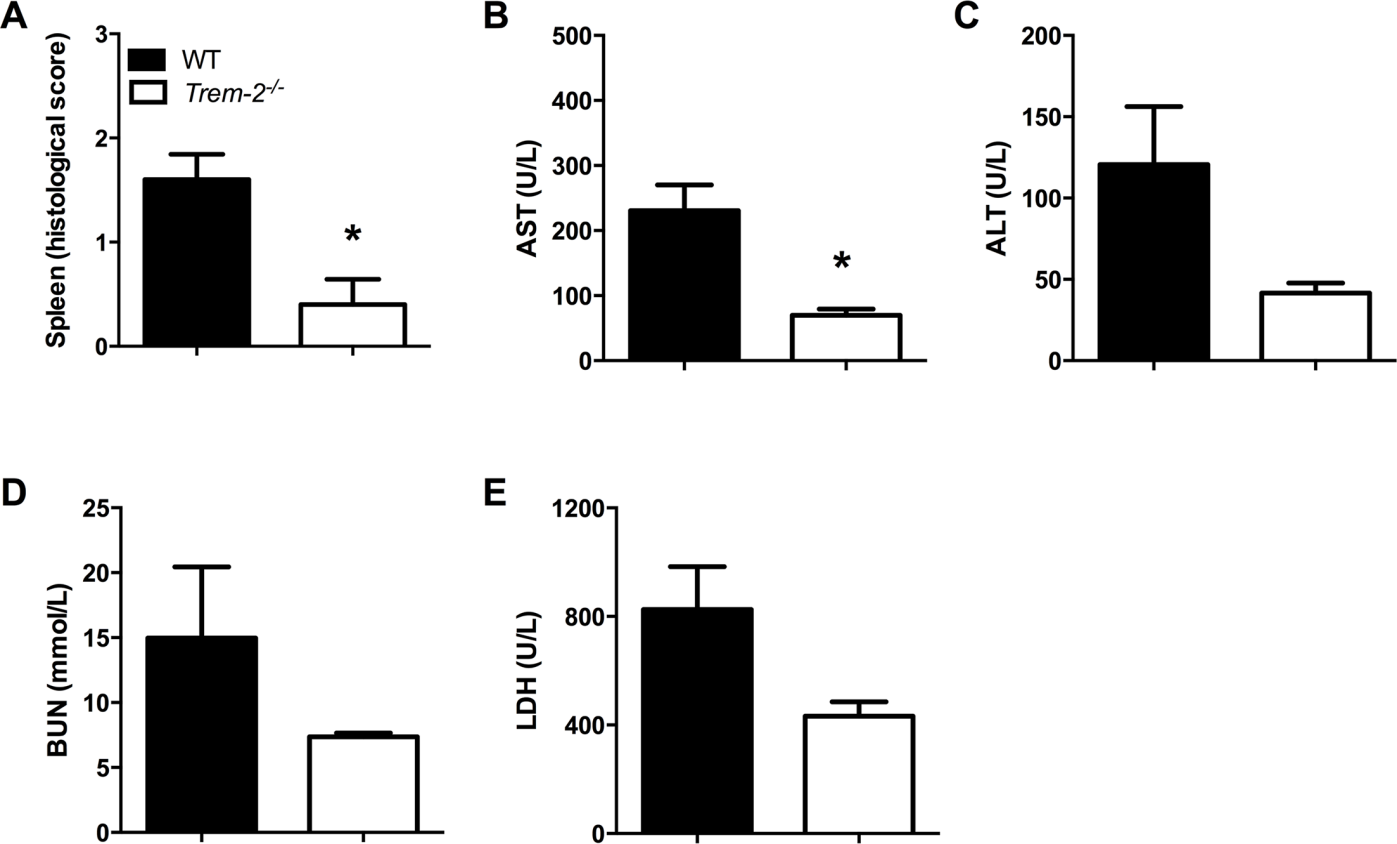
Reduced distant organ damage in *Trem-2*^*-/-*^ mice. At 72h post-infection with 5 x 102 CFU *B*. *pseudomallei* intranasally splenic injury (*A*) in WT (black bars) and *Trem-2*^*-/-*^ mice (white bars) was quantified as described in the Methods section. Plasma levels of aspartate transaminase (AST; *B*), alanine transaminase (ALT; *C*), Lactate dehydrogenase (LDH; *D*) and blood urea nitrogen (BUN; *E*) in WT and *Trem-2*^*-/-*^ mice were determined. Data are expressed as mean ± SEM. n = 5–6 mice per group per time point. *P < 0.05 (Mann-Whitney *U* test).

To evaluate the role of TREM-2 in the systemic inflammatory response, we determined plasma cytokine levels 72h post-infection with *B*. *pseudomallei*. Consistent with the lower pulmonary cytokine levels and bacterial loads, we found that the plasma levels of TNF-α, IL-6, IL-1β, MCP-1, IL-10, IFN-γ and KC were all significantly reduced in *Trem-2*^*-/-*^ mice compared to WTs (*P*<0.01–0.05, Table 1). Furthermore, we obtained spleen pathology scores and performed routine clinical chemistry tests to evaluate hepatic, renal and systemic injury. In line with the observed decreased splenic bacterial loads, *Trem-2*^*-/-*^ mice showed less inflammation compared to WT mice 72h after inoculation with *B*. *pseudomallei* (*P*<0.05; Fig 4A). Plasma AST levels of *Trem-2*^*-/-*^ mice were decreased when compared to controls 72h post-infection, reflecting decreased hepatocellular injury in these animals (*P*<0.05; Fig 4B). Consistently, we observed a trend towards lower ALT, BUN and LDH levels in *Trem-2*^*-/-*^ mice compared to controls suggesting less organ damage respectively (Fig 4C–4E).

### Lack of TREM-2 leads to a reduced inflammatory response ex vivo, but does not impact on phagocytosis of *B*. *pseudomallei* by macrophages

**Fig 5:**
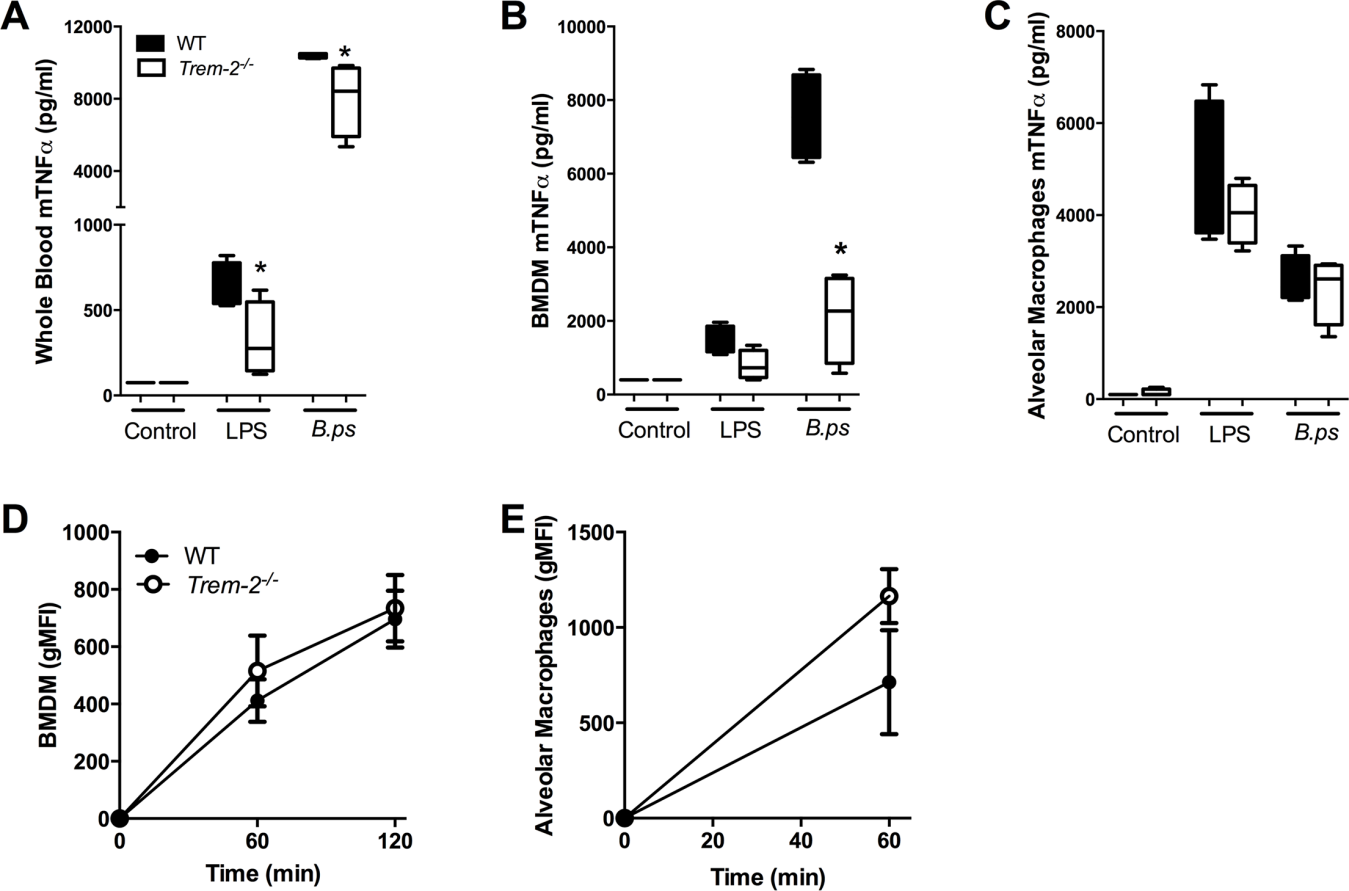
TREM-2 deficiency reduces cellular responsiveness *ex vivo*. Whole blood **(***A*), bone marrow derived macrophages (BMDM; *B*) and alveolar macrophages (AM; *C*) of WT (black bars) and *Trem-2*^*-/-*^ mice (white bars) were stimulated with medium, *E*.*coli* LPS (100 ng/ml) or heat-inactivated *B*. *pseudomallei* (107 CFU/ml or MOI of 50). Supernatant was collected after 20 h of stimulation and assayed for TNF-α. In addition, WT and *Trem-2*^*-/-*^ BMDM **(***D***)** and AM **(***E***)** were incubated at 37°C with FITC labeled heat-inactivated *B*. *pseudomallei* after which time-dependent phagocytosis was determined. Data are presented as mean ± SEM and are representative of two or three independent experiments. n = 4 or 8 per group. * *P*< 0.05 (Mann-Whitney *U* test).

Having established that TREM-2 plays an important deleterious role during experimental melioidosis and is involved in the inflammatory response, we next assessed what cells are responsible for these effects. It is known that blood monocytes, alveolar macrophages (AM) and BMDM express TREM-2 [25], therefore we harvested these cells and first stimulated them overnight with the TLR4-ligand LPS and *B*. *pseudomallei*. We found a clear trend towards lower TNF-α levels when whole blood, AM or BMDM of *Trem-2*^*-/-*^ mice were stimulated with LPS (Fig 5A–5C). This effect was even more pronounced after stimulation with *B*. *pseudomallei*: the TNF-α response of whole blood and BMDM derived from TREM-2 deficient mice was significantly reduced compared to controls (*P*<0.05; Fig 5A and 5B). Considering TREM-2’s known phagocytic properties [24, 25] and the observed lower local and systemic bacterial loads in TREM-2-deficient mice, we determined the phagocytic capacity of AM and BMDM harvested from WT and *Trem-2*^*-/-*^ mice. Despite a trend towards enhanced phagocytosis of FITC-labelled *B*. *pseudomallei* by TREM-2 deficient macrophages, no significant differences were found (Fig 5D and 5E). In line, TREM-2 did not impact on the intracellular killing of *B*. *pseudomallei* by BMDM (S1 Fig).

### Limited role of TREM-1/3 in the host defense during experimental melioidosis

**Fig 6:**
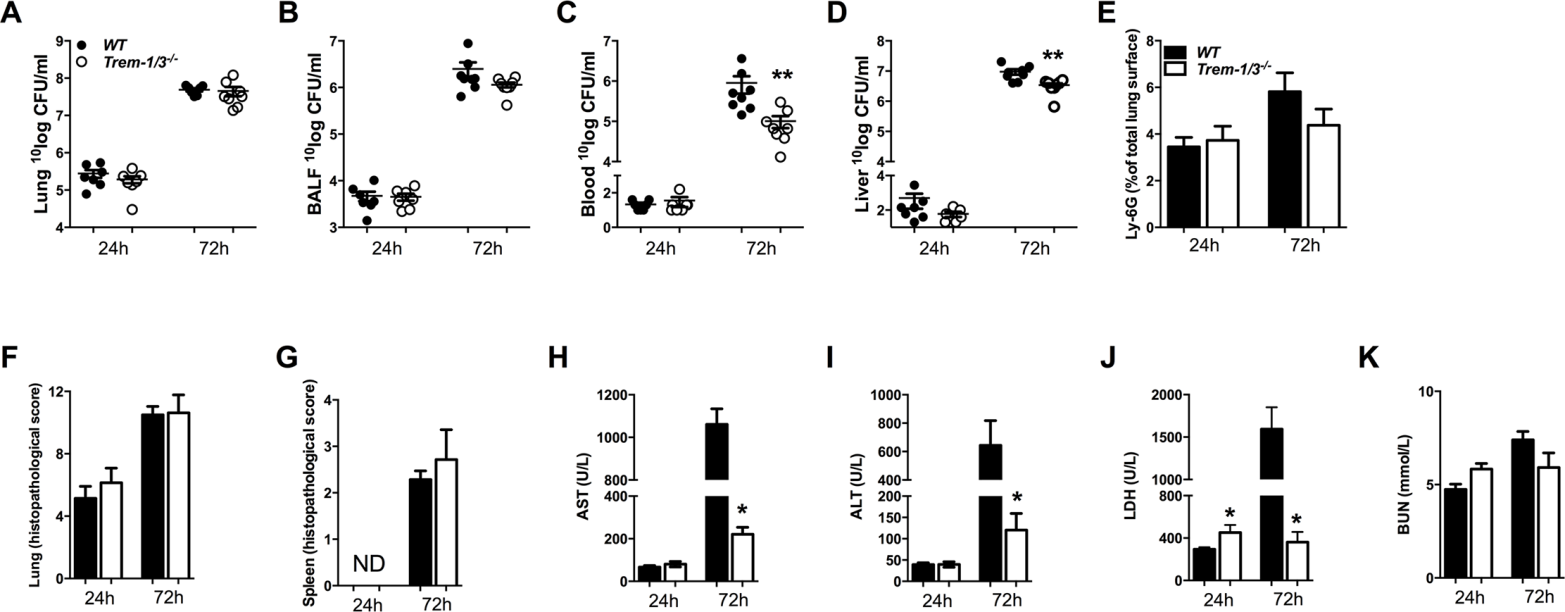
Effect of TREM-1 deficiency on bacterial clearance, pulmonary neutrophil influx and organ damage during experimental melioidosis. WT (closed circles/black bars) and *Trem-1/3*^*-/-*^ mice (open circles/ white bars) were intranasally infected with 5 x 102 CFU of *B*. *pseudomallei* and sacrificed 24 and 72 h post-infection, followed by determination of bacterial loads in lung homogenate **(***A***),** BALF **(***B***),** blood **(***C***)** and liver **(***D***).** Neutrophil influx as determined by % Ly6G positive surface of lung slides was calculated for WT and *Trem-1/3*^*-/-*^ mice **(***E***).** Lung **(***F***)** and spleen **(***G***)** pathology was scored as described in the Methods section. Aspartate transaminase (AST; *H***),** alanine transaminase (ALT; *I*), lactate dehydrogenase (LDH; *J***)** and blood urea nitrogen (BUN; *K*) were measured as a marker for end organ damage. Data are expressed as mean ± SEM. n = 7–8 mice per group. *P < 0.05; **P< 0.01 (Mann-Whitney *U* test).

In a final set of experiments we studied the role of TREM-1 in the host defense against *B*. *pseudomallei* using *Trem-1/3*^*-/-*^ mice. In contrast to the data derived from *Trem-2*^*-/-*^ mice, no differences in bacterial counts in lung or BALF were observed between *B*. *pseudomallei*-challenged *Trem-1/3*^*-/-*^ and WT mice (Fig 6A and 6B). In line, TREM-1 deficiency did not impact on lung pathology and cytokine levels, except for decreased KC levels, which did not influence pulmonary neutrophilic content as determined by Ly6-stainings (Fig 6E and 6F, Table 2). However, TREM-1 did influence bacterial dissemination as bacterial loads in blood and liver were significantly decreased in *Trem-1/3*^*-/-*^ mice compared to WTs 72h after infection (*P*<0.01; Fig 6C and 6D). We next evaluated TREM-1’s role in systemic inflammation and end organ damage. At 72h post-infection, the levels of key regulatory cytokines in the systemic compartment (TNF-α, IL-6, IL-10, MCP-1 and IFN-γ) did not differ between *Trem-1/3*^*-/-*^ mice and WT (Table 2). Induced pathology of the spleen (Fig 6G) was similar in *Trem-1/3*^*-/-*^ and WT mice. In correspondence with the lower hepatic bacterial counts at 72h, we found lower levels of the hepatocellular injury markers AST and ALT levels in *Trem-1/3*^*-/-*^ mice compared to WT mice (Fig 6H and 6I). LDH levels, reflecting general organ injury, were elevated in *Trem-1/3*^*-/-*^ mice at 24 h, while they were reduced compared to their WT counterparts at 72h post-infection (*P*<0.05; Fig 6J). No difference in plasma BUN levels was observed between mice strains (Fig 6K).

**Table 2:** Cytokine responses in lung homogenates, BALF and plasma of WT and *Trem-1/3*^*-/-*^ mice during experimental melioidosis.

	T = 24h		T = 72h	
	WT	*Trem-1/3*^*-/-*^	WT	*Trem-1/3*^*-/-*^
**pg/ml**	**Lung homogenate**			
**TNF-α**	1406 ± 220	1227 ± 215	2135 ± 312	2312 ± 386
**IL-6**	2380 ± 293	2913 ± 353	17694 ± 3121	29910 ± 4668
**KC**	18817 ± 5116	14277 ± 1981	71606 ± 6071	69866 ± 8092
**pg/ml**	**BALF**			
**TNF-α**	883 ± 100	808 ±95	2472 ± 359	3624 ± 831
**IL-6**	1675 ± 285	654 ± 97**	6041 ± 942	9449 ± 1914
**KC**	992 ± 183	711 ± 113	19039 ± 1614	12251 ± 1678*
**pg/ml**	**Plasma**			
**TNF-α**	12 ± 1	9 ±1	616 ± 160	307 ± 77
**IL-6**	143 ± 36	133 ± 19	2868 ± 818	2695 ± 313
**IL-10**	8 ± 2	20 ± 6	199 ± 77	81 ± 36
**MCP-1**	263 ± 63	92 ± 25*	2335 ± 94	2975 ± 226
**IFN-γ**	28 ± 4	26 ± 4	1606 ± 445	1275 ± 242

Cytokine levels in plasma, lung homogenate and broncho-alveolar fluid (BALF) were measured after intranasal infection with 5 x 102 CFU wild-type *B*. *pseudomallei*. Wild-type (WT) and *Trem-1/3*^*-/-*^ mice were sacrificed 24 and 72 h after infection. Data are represented as means ± SEM (n = 7 or 8/group per time point). TNF-α = Tumor necrosis factor-α; IL = Interleukin; MCP-1 = Monocyte Chemoattractant Protein-1; KC = Keratinocyte Chemoattractant; IFN-γ = Interferon-γ

* *P* < 0.05

** *P* < 0.01.

### TREM-1 deficiency does not impact on ex vivo cytokine responsiveness and phagocytosis nor intracellular killing of *B*. *pseudomallei*

**Fig 7:**
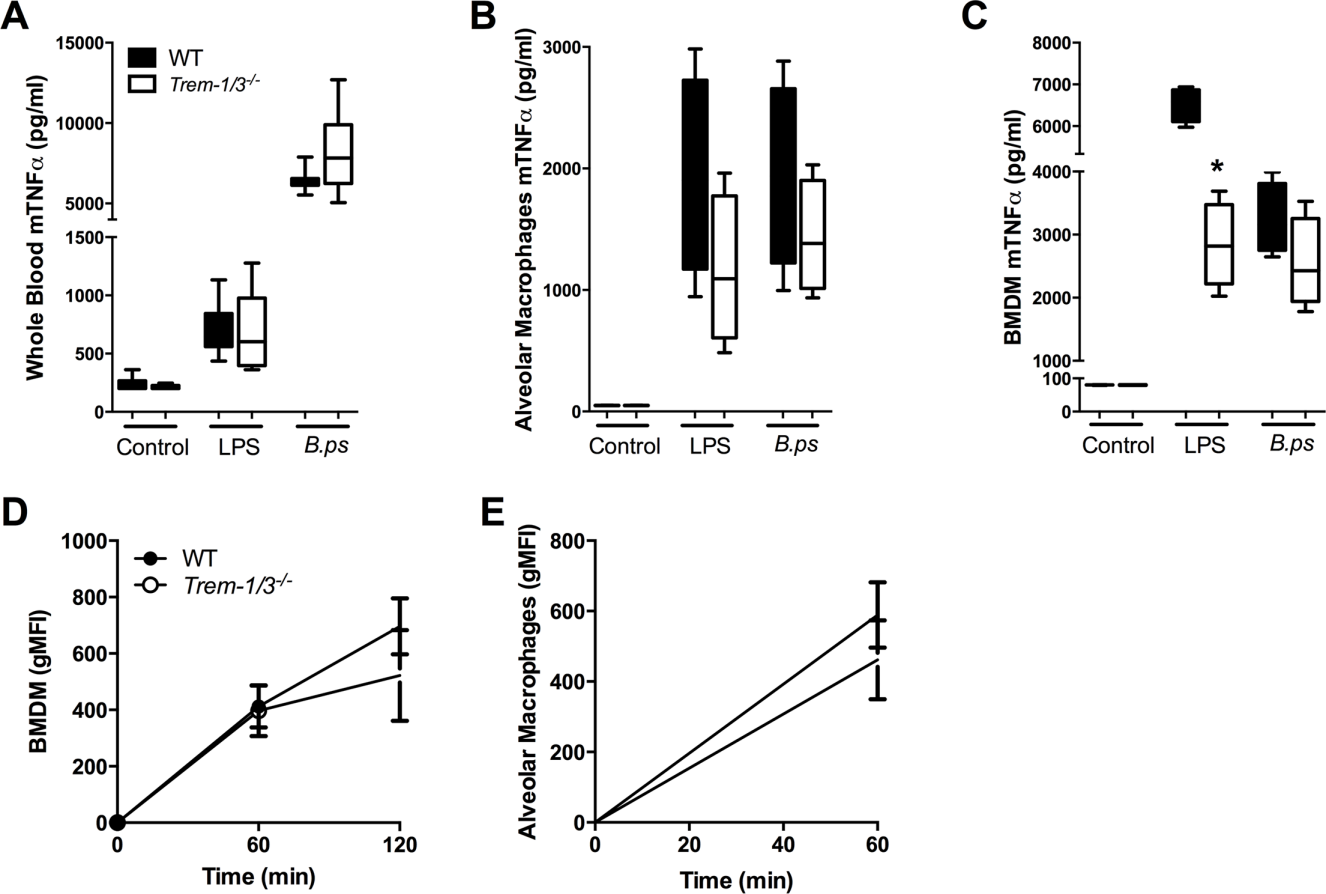
No effect of TREM-1 deficiency on the cellular responsiveness and phagocytosis or intracellular killing of *B*. *pseudomallei*. Whole blood (*A*), bone marrow derived macrophages (BMDM; *B*) and alveolar macrophages (AM; *C***)** of WT and *Trem-1/3*^*-/-*^ mice were stimulated with medium, *E*.*coli* LPS(100 ng/ml) or heat-inactivated wild type *B*. *pseudomallei* (107 CFU/ml at a MOI of 50). TNF-α levels were measured in the supernatant obtained after 20 h of stimulation. BMDM (*D*) and AM (*E*) of WT and *Trem-1/3*^*-/-*^ mice were incubated at 37°C with FITC labeled heat-inactivated *B*. *pseudomallei* after which time-dependent phagocytosis was determined. Data are expressed as mean ± SEM and are representative of two or three independent experiments. n = 4 or 8 (for the whole blood assay) per group. *P< 0.05 (Mann-Whitney *U* test).

TREM-1 is abundantly expressed on monocytes and macrophages following exposure to *B*. *pseudomallei* [31]. In line with previous findings [11], *Trem-1/3*^*-/-*^ BMDM produced less TNF-α in response to LPS stimulation (*P*<0.05; Fig 7B). Surprisingly, no differences in cellular responsiveness were found between AM and whole blood derived from WT and *Trem-1/3*^*-/-*^mice (Fig 7A–7C). Lastly, we wished to determine whether TREM-1 contributes to phagocytosis and/or killing of *B*. *pseudomallei*. No differences in phagocytic and killing capacities between WT and TREM-1 deficient BMDM were observed (Fig 7D and 7E).

## Discussion

TREM-1 and TREM-2 are innate immune receptors that have demonstrated to either amplify or regulate TLR and NLR signaling after recognition of pathogen-associated molecular patterns. Our study is the first to examine the role of both TREM-1 and TREM-2 during experimental melioidosis. We observed increased TREM-1 and TREM-2 expression during experimental melioidosis, both at the local site of infection and systemically. Subsequently, we found that TREM-2 impairs the host defense against murine *B*.*pseudomallei*-induced sepsis, as demonstrated by an improved survival of infected *Trem-2*^*-/-*^ mice as a direct result of diminished bacterial dissemination, decreased inflammation and less organ damage. Our *ex vivo* studies suggest that the protective effect of TREM-2 deficiency in part results from the diminished capacity of TREM-2-deficient macrophages to elicit a pro-inflammatory response which is an important contributor to organ injury in the event of sepsis. TREM-1 was also found to play a detrimental role during *B*. *pseudomallei* infection, which is in line with our earlier finding that blocking TREM-1 could improve survival during melioidosis [31]. However when compared to TREM-2 the role of TREM-1 in the host response against *B*. *pseudomallei* seems to be limited.

Previous studies have demonstrated that soluble TREM-1 levels are up-regulated in plasma of patients with sepsis, pneumonia and melioidosis [31, 41, 42]. In addition, it is known that surface TREM-1 expression is increased on monocytes of melioidosis patients [31]. However, soluble TREM-1 levels in septic patients do not always correlate to the expression of membrane-bound TREM-1 on different myeloid cell types [31, 43]. Less is known about the kinetics of TREM-2 expression during infection. A recent study demonstrated that during sepsis TREM-2 expression on ascites-retrieved cells of patients with abdominal sepsis was increased [27]. Correspondingly, TREM-2 was up-regulated on AM of mice infected with *S*. *pneumoniae* [25]. In line with these earlier studies, we now show that both TREM-1 and TREM-2 mRNA expression is elevated in lung and liver tissue of mice infected with *B*. *pseudomallei*. Further research however is warranted to study the cell surface protein expression of TREM-2 on neutrophils and macrophages during melioidosis.

The *in vivo* role of TREM-2 in infectious diseases remains ill defined. In a model studying *P*. *aeruginosa* keratitis TREM-2 deficiency increased corneal bacterial loads [44]. More recently, Chen *et al*. demonstrated that TREM-2 is required for efficient bacterial clearance in a murine polymicrobial sepsis model using a TREM-2 blocking recombinant protein [27]. In the same study it was shown that administration of TREM-2 overexpressing bone marrow derived myeloid cells improved survival during polymicrobial sepsis, but not endotoxaemia [27]. In sharp contrast, Gawish et al. demonstrated a beneficial effect of TREM-2 deficiency during endotoxaemia [45]. The same group also observed a survival benefit of *Trem-2*^*-/-*^ mice during *S*. *pneumoniae* pneumonia [25], while no effect on mortality of TREM-2 deficiency was seen during *E*. *coli* sepsis [45]. To evaluate how TREM-2 deficiency led to increased clearance of *B*. *pseudomallei*, we assessed the functional roles of macrophages that express TREM-2 [19, 25]. TREM-2 is known to be involved in direct killing [27, 44] and phagocytosis of bacteria by macrophages [24, 25]. Interestingly, we did not find impaired bacterial killing or phagocytosis of *B*. *pseudomallei* by BMDM or AM of *Trem-2*^*-/-*^ mice. Several characteristics of this facultative intracellular bacterium when compared to other bacteria might in part explain these discrepancies; *B*. *pseudomallei* is capable of invading both phagocytic and non-phagocytic cells [46] and circumvents intracellular defense mechanisms efficiently in order to replicate and spread to adjacent cells [47, 48].

TREM-2 is traditionally regarded as a negative regulator of the *in vitro* inflammatory response in response to TLR-ligands [19, 21, 45] In contrast, our study now demonstrates that TREM-2 deficiency leads to a reduced inflammatory response to *B*. *pseudomallei* both *ex vivo* and *in vivo* suggesting that TREM-2’s role during inflammation may not be that upfront. This is in line with recent studies investigating the role of TREM-2 in models of pneumococcal *pneumonia* [25], post-stroke inflammation [49] and DSS-induced colitis [50]. Different elements, can explain these inconsistencies: differences in mice strains used (BALB/C versus C57Bl/6), different experimental murine models (e.g. caecal ligation and puncture (CLP)- model versus a intranasal inhalation model for sepsis), differences in TREM-2 blockade (e.g. by using TREM-2 deficient mice or TREM-2 antibodies) and lastly the difference of an *in vitro* approach in contrast to our *ex vivo* cellular challenge model. Interestingly, a recent study showed augmented inflammation by TREM-2 deficient peritoneal macrophages in response to LPS [45], while the same group observed the reversed phenotype in alveolar macrophages [25], underlining possible cell-specific functions of TREM-2. Of importance, neutrophil recruitment to the lung, an important defense mechanism during melioidosis [32, 51], was reduced in *Trem-2*^*-/-*^ mice during experimental melioidosis as determined by Ly6-staining. This may be a potential result of the decreased inflammatory response and production of chemokines following infection. In this respect, it is noteworthy, that IL-1β–which we and others have shown to be involved in excessive deleterious neutrophil influx during experimental melioidosis [37, 52]—was also significantly reduced in *Trem-2*^*-/-*^ mice. No differences were observed in the influx of macrophages (S2 Fig). Excessive inflammation and neutrophil influx and activation can lead towards multi-organ failure [53], which is almost universally seen in lethal cases of melioidosis. Distant organ injury was significantly reduced in *Trem-2*^*-/-*^ mice, potentially as a result of a reduced influx of inflammatory cells. *Trem-2*^*-/-*^ mice displayed an evidently reduced inflammatory response, which resulted in a strong survival benefit. In addition, it is well known that *B*. *pseudomallei* can replicate intracellularly [28], and neutrophils may act as its permissive host cell [52]. We could therefore hypothesize that the anti-inflammatory phenotype and the reduced bacterial loads seen in TREM-2 deficient mice are a result of decreased intracellular bacterial replication at the infection site, due to reduced neutrophilic influx.

Taken together, during melioidosis, TREM-2 deficiency resulted in a restricted inflammatory response, thereby decreasing organ damage and mortality. Future research should focus on the potential of anti-TREM-2 treatment of *B*.*pseudomallei*-infected mice.

TREM-1 amplifies TLR-responses and therefore might dangerously enhance the inflammatory response to bacterial infection [18]. Controversial results have been found on the role of TREM-1 during bacterial infection. TREM-1 deficiency has shown to be detrimental during endotoxaemia [17] and polymicrobial sepsis [12, 54], while in contrast, moderate levels of TREM-1 can improve survival during polymicrobial sepsis, but not endotoxaemia [55]. Blockade of TREM-1 with a peptide called LP17 could partially protect mice from *B*. *pseudomallei* induced lethality [31]. In this study however, we observed, using the same infection model, that survival of *B*. *pseudomallei*-infected TREM-1-deficient mice was similar to WTs. This might be explained by the fact that these mice were completely TREM-1-deficient and in addition lacked TREM-3, a DAP12-coupled activating receptor on murine macrophages, which supposedly acts as an activating receptor [56]. In contrast, in humans TREM-3 is a pseudogene [56]. However, since DAP12 is known to both potentiate and attenuate TLR-signaling, it is perhaps not surprising that the net-effect on bacterial clearance of *B*. *pseudomallei* is not affected.

TREM-1 has other functions next to TLR-signaling enhancement, such as phagocytosis and the production of reactive oxygen species [57]. Furthermore, TREM-1 has been recently linked to trans-epithelial migration of neutrophils after infection with *P*. *aeruginosa* [15]. Blocking TREM-1 completely could therefore interfere with these important antibacterial mechanisms. We did not find a role for TREM-1 in the killing or phagocytosis of *B*. *pseudomallei*, which is in line with the fact that TREM-1/3 deficiency in neutrophils neither impacts on bacterial killing, phagocytosis and chemotaxis of *P*. *aeruginosa* [15]. This suggests that other phagocytic receptors on leukocytes are more important for the efficient eradication of *B*. *pseudomallei* [38, 58, 59].

Murine models like the one used here, which make use of relatively young mice exposed to an intranasal bacterial inoculum, do show inter-experiment variation, as reflected by differences in bacterial dissemination and as a result inflammation at the latter time-points before mice will succumb to infection. In addition, caution is needed when extrapolating data from murine experiments to human disease.”

Taking these precautions into mind, we here demonstrate that murine melioidosis is associated with increased TREM-1 and -2 expression. TREM-2 deficiency is beneficial during experimental Gram-negative sepsis caused by a clinical relevant pathogen, resulting in lower bacterial loads, reduced organ damage, decreased inflammation and improved survival. When compared to TREM-2, TREM-1 plays a limited detrimental role during experimental melioidosis. These results provide new information on the expression and function of TREM-2 during melioidosis and may demonstrate its potential therapeutic usefulness.

## Supporting Information

S1 Appendix Supplemental materials and methods. (DOC)

S1 Fig Intracellular killing of *B*. *pseudomallei* by BMDM is not impaired by TREM-2 deficiency. WT and *Trem-2*^*-/-*^ BMDM were incubated at 37°C with live *B*. *pseudomallei* after which time-dependent intracellular killing was determined. Data are presented as mean ± SEM and are representative of two independent experiments. n = 6 per group (Mann-Whitney U test).
(TIF)

S2 Fig Similar macrophage influx in BALF of WT and *Trem-2*^*-/-*^ during experimental melioidosis. Macrophage influx in broncho-alveolar lavage fluid (BALF) was determined 72h post-infection with 5 x 102 CFU *B*. *pseudomallei* in wild-type (WT; black circles) and *Trem-2*^*-/-*^ mice (white circles). Data are presented as mean ± SEM n = 5–6 mice/group (Mann- Whitney U test).
(TIF)
