# Supplementary material for: Triggering Receptor Expressed on Myeloid Cells (TREM)-2 Impairs Host Defense in Experimental Melioidosis
Source: PLoS Negl Trop Dis. 2016 Jun 2;10(6):e0004747. doi: 10.1371/journal.pntd.0004747 (PMC4890812; doi:10.1371/journal.pntd.0004747)
Supplement: S1 Appendix — (DOC) [file pntd.0004747.s001.doc]

# S1 Appendix:

# Triggering receptor on myeloid cells (TREM)-2 impairs host defenSe in Gram-negative sepsis (melioidosis)

*Tassili A. F. Weehuizen ^1,2^*, Tijmen J. Hommes ^1,2^, Jacqueline M. Lankelma ^1,2^, Hanna K. de Jong^1,2^, Joris. J.T.H. Roelofs^3^, Alex F. de Vos^1,2^, Marco Colonna^4^, Tom van der Poll^1,2,5^, W. Joost Wiersinga^1,2,5^*

^1^Center for Infection and Immunity Amsterdam (CINIMA), Academic Medical Center, Amsterdam, 1105 AZ, the Netherlands

^2^Center for Experimental and Molecular Medicine (CEMM), Academic Medical Center, Amsterdam, 1105 AZ, the Netherlands

^3^Department of Pathology, Academic Medical Center, Amsterdam, 1105 AZ, the Netherlands

^4^Department of Pathology, Washington University in St. Louis, 63110, MO, USA

^5^Department of Medicine, Division of Infectious Diseases, Academic Medical Center, Amsterdam, 1105 AZ, the Netherlands

**MATERIALS AND METHODS**

##### Mice

TREM-1 and -2 deficiency was confirmed by polymerase chain reaction.

All mice were kept at the animal care facility of the Academic Medical Center (University of Amsterdam) with unlimited access to food and water.

##### Experimental infection

10 μl of a frozen aliquot of *B.pseudomallei* was pipetted into 50 ml Luria broth (Sigma-Aldrich) for overnight incubation at 37 °C in a 5% CO2 incubator. Thereafter, 1 ml of the bacterial suspension was transferred to fresh Luria broth and grown for approximately 3h to midlogarithmic phase. Bacteria were harvested by centrifugation at 1500xg for 15 min, washed, and resuspended in sterile PBS 1x. Concentrations were determined by plating serial 10-fold dilutions on blood agar plates. Pneumonia was induced by intranasally inoculating mice with 50 μl containing 0.5 x 10^2^ CFU of *B.pseudomallei*.

For the infection procedure, mice were lightly anesthetized by inhalation of isoflurane.

24 and 72 hours (h) after infection, mice were sacrificed under intraperitoneal anesthesia containing ketamin (Eurovet Animal Health, Bladel, The Netherlands) and medetomidin (Pfizer Animal Health Care, Capelle aan den IJssel, The Netherlands). Lungs, liver and spleen were harvested and homogenized at 4°C in 4 volumes of sterile saline using a tissue homogenizer (Biospec Products, Bartlesville, OK). Broncho-alveolar lavage (BAL) was performed by instilling two 0.5 ml aliquots of sterile saline into the airway through cannulation of the trachea with a sterile 22-gauge Abbocath-T catheter (Abbott, Sligo, Ireland). Serial dilutions of organ homogenates, BAL fluid (BALF) and blood were plated on blood agar plates and incubated at 37°C at 5% CO_2_ overnight. Lung homogenates were diluted 1:1 in Greenberger lysis buffer containing 300 mM NaCl, 30 mM Tris, 2 mM MgCl_2_, 2 mMCaCl_2_, 1% Triton X-100 and protease inhibitors (Roche, Indianapolis, IN), incubated at 4°C for 30 min and centrifuged at 1730x*g* at 4°C for 10 min. Supernatants of lung and BALF and plasma were sterilized using 0.22 μm pore-size filters (Millipore, Billerica, MA) and stored at -20°C until further analysis.

***Trem-1 and Trem-2 expression***

##### For RT-PCR on murine lung and liver tissues the following conditions were used: 6 min at 95°C, followed by 45 cycles of amplification (95°C for 10 s, 60°C for 5 s, and 72°C for 15 s). Data were analysed by the comparative Ct method using GAPDH as the housekeeping gene and presented as a fold induction compared to gene-expression in naïve mice. The forward primer used for mTREM-1 was (5’- 3’) TGTTGTGCTCTTCCATCCTG and the reverse was (5’- 3’) GGGATCGGGTTGTAGTTGTG. For mTREM-2 these were (5’- 3’) AGATGACCAAGATGCTGGAGA and (5’- 3’) ATGCTGGCTGCAAGAAACTT respectively. For mGAPDH the forward primer (5’- 3’) ATGGCCTTCCGTGTTCCTAC and reverse primer (5’- 3’) AGGAGACAACCTGGTCCTCA were used. Oligonucleotides were purchased from Eurogentec (Maastricht, The Netherlands).

##### Assays

##### Plasma mouse tumour necrosis factor-α (TNF-α), interleukin (IL)-6, IL-10, IL-12p70, monocyte chemoattractant protein-1 (MCP-1) and interferon (IFN)-γ were measured by cytometric bead array multiplex assay (BD Biosciences, San Jose, CA, USA). Keratinocyte chemoattractant (KC), TNF-α, IL-1ß and IL-6 levels in lung and BALF were measured by ELISA (R&D systems, Minneapolis, MN). Alanine aminotransaminase [1] and aspartate aminotransferase (AST), blood urea nitrogen (BUN) and lactate dehydrogenase (LDH) levels were determined with the Cobas 8000 module c702 (Roche Diagnostics).

***(Immuno)histology***

Lungs, livers and spleens for histology were harvested after infection, fixed in 100% formalin and embedded in paraffin. Sections of 4 μm were stained with haematoxylin–eosin (HE), and analysed by a pathologist who was blinded to the groups [2-4]. Lung pathology was scored based on affected surface, necrosis and/or abscess formation, interstitial inflammation, endothelialitis, bronchitis, edema, thrombus formation, and pleuritis. Each parameter was graded on a scale of 0 to 4. The total “mean histological score” was expressed as the sum of the scores for each parameter, (maximum of 32). Spleen and liver sections were scored on inflammation, necrosis/abscess formation, and thrombus formation using the scale given above (maximum score of 12).

Granulocyte (Ly6G) staining was performed as previously described [5] The Ly6G antibody detects Ly6G, formerly known as myeloid differentiation antigen Gr-1, a 21–25- kDa GPI-anchored protein that can be found on granulocytes, including neutrophils and eosinophils. Slides were counterstained with methylgreen (Sigma-Aldrich, St. Louis, MO, USA). The total tissue area of the Ly-6-stained slides was scanned with a slide scanner (Olympus dotSlide, Tokyo, Japan) and the obtained scans were exported in TIFF format for digital image analysis. The digital images were analyzed with IMAGEJ (version 1.47v, National Institutes of Health, Bethesda, MD, USA) and the immunopositive (Ly6 +) area was expressed as the percentage of the total lung surface area.

***Whole blood and macrophage stimulation***

Whole blood, alveolar macrophages (AM) and bone-marrow derived macrophages (BMDM) were harvested from naïve WT and *Trem1/3^-/-^* and *Trem-2^-/-^* mice as described [3, 6, 7]. 50 µl of whole blood was stimulated with 50 µl of stimulus in a 96-well V-bottom plate (Greiner Bio-one, Alphen a/d Rijn, the Netherlands), either plain RPMI 1640 (Life Technologies, Bleiswijk, The Netherlands), *B. pseudomallei* 2 x 10^7^ CFU/ml or lipopolysaccharide (LPS) of *E.coli* O111:B4 (end concentration of 100 ng/ml) (Invitrogen, San Diego, CA, USA) overnight at 37 °C and 5% CO_2_ air after which supernatant was harvested and stored at -20°C until assayed. AM (5 x 10^4^/well) and BMDM (1 x 10^5^/well) were stimulated in a 96-well flat bottom plate (Greiner) either with RPMI + FCS 10% (Life technologies) *B. pseudomallei* (MOI 50) or LPS of *E.coli* (end concentration 100 ng/ml) at 37 °C and 5% CO_2_ air overnight after which the supernatants were stored at -20°C until assayed.

**REFERENCES**

1. Centers for Disease C, Prevention DoH, Human S. Possession, use, and transfer of select agents and toxins; biennial review. Final rule. Federal register. 2012;77(194):61083-115. PubMed PMID: 23038847.

2. Hommes TJ, Hoogendijk AJ, Dessing MC, Van't Veer C, Florquin S, Colonna M, et al. Triggering receptor expressed on myeloid cells-1 (TREM-1) improves host defence in pneumococcal pneumonia. The Journal of pathology. 2014;233(4):357-67. doi: 10.1002/path.4361. PubMed PMID: 24752755.

3. Wiersinga WJ, Wieland CW, Dessing MC, Chantratita N, Cheng AC, Limmathurotsakul D, et al. Toll-like receptor 2 impairs host defense in gram-negative sepsis caused by Burkholderia pseudomallei (Melioidosis). PLoS medicine. 2007;4(7):e248. doi: 10.1371/journal.pmed.0040248. PubMed PMID: 17676990; PubMed Central PMCID: PMC1950213.

4. Wiersinga WJ, de Vos AF, de Beer R, Wieland CW, Roelofs JJ, Woods DE, et al. Inflammation patterns induced by different Burkholderia species in mice. Cellular microbiology. 2008;10(1):81-7. doi: 10.1111/j.1462-5822.2007.01016.x. PubMed PMID: 17645551.

5. Kager LM, Wiersinga WJ, Roelofs JJ, Meijers JC, Zeerleder SS, Esmon CT, et al. Endogenous protein C has a protective role during Gram-negative pneumosepsis (melioidosis). Journal of thrombosis and haemostasis : JTH. 2013;11(2):282-92. doi: 10.1111/jth.12094. PubMed PMID: 23216621.

6. Eske K, Breitbach K, Kohler J, Wongprompitak P, Steinmetz I. Generation of murine bone marrow derived macrophages in a standardised serum-free cell culture system. J Immunol Methods. 2009;342(1-2):13-9. doi: 10.1016/j.jim.2008.11.011. PubMed PMID: 19133267.

7. Koh GC, Weehuizen TA, Breitbach K, Krause K, de Jong HK, Kager LM, et al. Glyburide reduces bacterial dissemination in a mouse model of melioidosis. PLoS neglected tropical diseases. 2013;7(10):e2500. doi: 10.1371/journal.pntd.0002500. PubMed PMID: 24147174; PubMed Central PMCID: PMC3798430.
