# Supplementary figures and images for: Triggering Receptor Expressed on Myeloid Cells (TREM)-2 Impairs Host Defense in Experimental Melioidosis
Source: PLoS Negl Trop Dis. 2016 Jun 2;10(6):e0004747. doi: 10.1371/journal.pntd.0004747 (PMC4890812; doi:10.1371/journal.pntd.0004747)

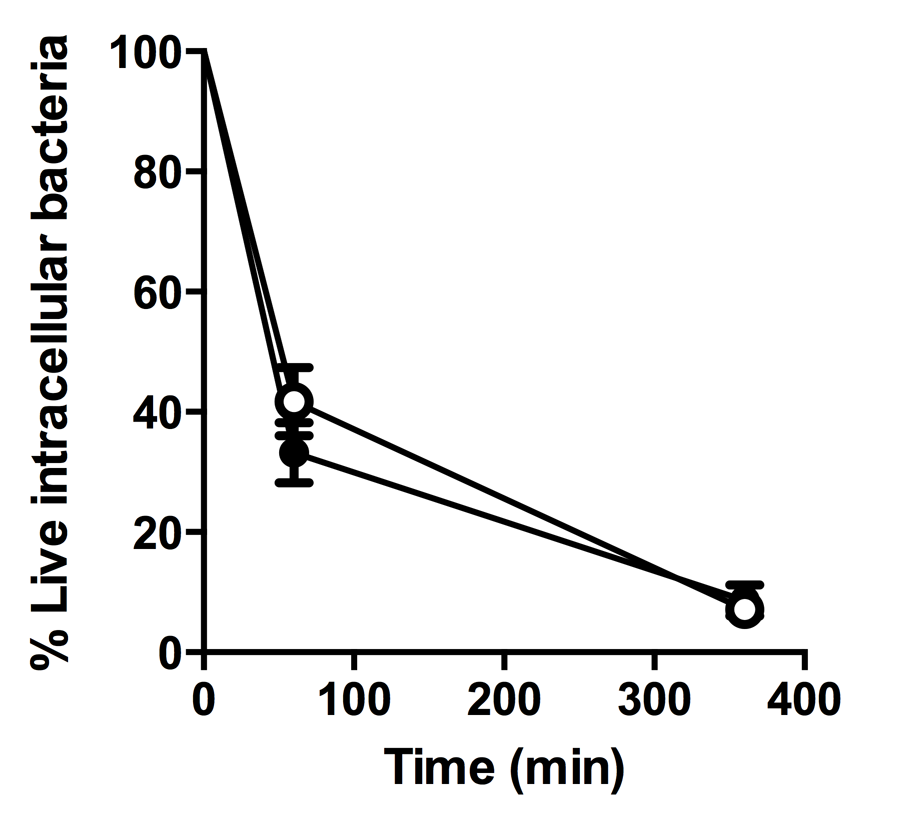

Supplement: S1 Fig — WT and Trem-2-/- BMDM were incubated at 37°C with live B. pseudomallei after which time-dependent intracellular killing was determined. Data are presented as mean ± SEM and are representative of two independent experiments. n = 6 per group (Mann-Whitney U test). (TIF) [file pntd.0004747.s002.tif]

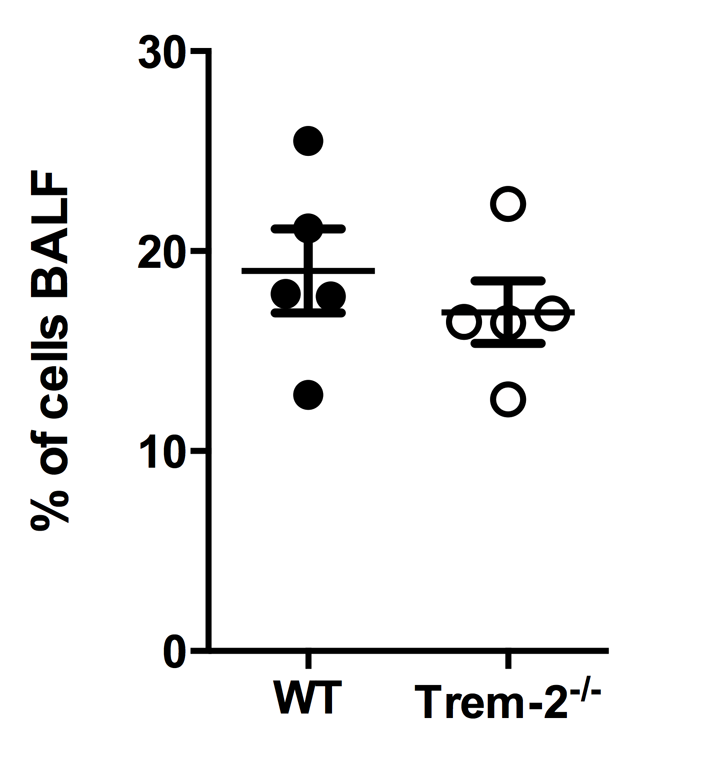

Supplement: S2 Fig — Macrophage influx in broncho-alveolar lavage fluid (BALF) was determined 72h post-infection with 5 x 10^2^ CFU B. pseudomallei in wild-type (WT; black circles) and Trem-2-/- mice (white circles). Data are presented as mean ± SEM n = 5–6 mice/group (Mann- Whitney U test). (TIF) [file pntd.0004747.s003.tif]
